# Supplementary material for: Observation of Rayleigh Optical Activity for Chiral Molecules: A New Chiroptical Tool
Source: J Phys Chem A. 2025 Dec 4;129(51):11884–7. doi: 10.1021/acs.jpca.5c05516 (PMC12746440; doi:10.1021/acs.jpca.5c05516)
Supplement: Supplementary file 1 [file jp5c05516_si_001.pdf]

Observation of Rayleigh Optical Activity for Chiral Molecules: A New Chiroptical Tool Supporting Information

Duncan McArthur,[†,⊥] Emmanouil I. Alexakis,[†,⊥] Andrew R. Puente,[‡] Rebecca McGonigle,[¶] Andrew J. Love,[§] Prasad L. Polavarapu,[‡] Laurence D. Barron,[‖] Lewis E. MacKenzie,[¶] Aidan S. Arnold,[†] and Robert P. Cameron[*,†]

†*SUPA and Department of Physics, University of Strathclyde, Glasgow, G4 0NG, UK*

‡*Department of Chemistry, Vanderbilt University, Nashville, TN 37235, USA*

¶*Department of Pure and Applied Chemistry, University of Strathclyde, Glasgow, G1 1RD, UK*

§*James Hutton Institute, Invergowrie, Dundee DD2 5DA, UK*

‖*Department of Chemistry, University of Glasgow, Glasgow G12 8QQ, UK*

⊥*These authors contributed equally.*

E-mail: robert.p.cameron@strath.ac.uk

Our raw measured values are

$$\Delta'(1S, 5S) = (+4.78 \pm 0.28) \times 10^{-4}$$

$$\Delta'(1R, 5R) = (-2.08 \pm 0.29) \times 10^{-4},$$

where we have quoted the random uncertainties. To ensure a fair comparison with theory,

we correct these values as follows for small enantiomeric imbalances in our samples, which we attribute to thermal racemization; slight rotation of the incident light as it propagates through the samples, which introduces isotropic Rayleigh scattered light into the detected signals; and a small enantiomer-independent offset in our instrument. The offset is always small in magnitude ($\lesssim 1 \times 10^{-4}$) and can be tuned to zero precisely via judicious choice of integration boundaries in our data analysis, however we have refrained from doing so here in the interests of transparency.

We take the raw measured values $\Delta'(1S, 5S)$ and $\Delta'(1R, 5R)$ to be related to the corrected measured values $\Delta(1S, 5S) = -\Delta(1R, 5R)$ as

$$\Delta'(1S, 5S) = \frac{ee(1S, 5S)}{q(1S, 5S)}\Delta(1S, 5S) + \Delta'_{\text{offset}}$$
$$\Delta'(1R, 5R) = \frac{ee(1R, 5R)}{q(1R, 5R)}\Delta(1R, 5R) + \Delta'_{\text{offset}},$$

where $ee(1S, 5S)$ and $ee(1R, 5R)$ are the enantiomeric excesses of the samples, $q(1S, 5S)$ and $q(1R, 5R)$ account for the isotropic Rayleigh scattered light, and Δ'_{offset} is the offset. Simple algebra then gives

$$\Delta(1S, 5S) = f[\Delta'(1S, 5S) - \overline{\Delta}']$$
$$\Delta(1R, 5R) = f[\Delta'(1R, 5R) - \overline{\Delta}'],$$

where

$$f = 2\left[\frac{ee(1S, 5S)}{q(1S, 5S)} + \frac{ee(1R, 5R)}{q(1R, 5R)}\right]^{-1}$$

is a magnitude rescaling factor and

$$\overline{\Delta}' = \frac{1}{2}\left[\Delta'(1S,5S) + \Delta'(1R,5R)\right]$$
$$= (+1.35 \pm 0.20) \times 10^{-4}$$

is the average raw measured value.

For enantiopure samples, we would expect the rotation angles of the incident light to be

$$\Delta\theta(1S,5S) = [\Delta\theta]_{\mathrm{D}}(1S,5S) l\rho(\lambda_{\mathrm{D}}/\lambda)^2$$
$$= (-5.332 \pm 0.022)^\circ$$
$$\Delta\theta(1R,5R) = [\Delta\theta]_{\mathrm{D}}(1R,5R) l\rho(\lambda_{\mathrm{D}}/\lambda)^2$$
$$= (+5.332 \pm 0.022)^\circ,$$

where $[\Delta\theta]_{\mathrm{D}}(1S,5S) = -[\Delta\theta]_{\mathrm{D}}(1R,5R) = (-50.70 \pm 0.05)^\circ\,\mathrm{dm}^{-1}\,\mathrm{g}^{-1}\,\mathrm{cm}^3$ are the specific rotations of the enantiomers of α-pinene at the sodium D line, $l = (0.1000 \pm 0.0001)\,\mathrm{dm}$ is the internal path length of the cuvettes, $\rho = (0.8580 \pm 0.0005)\,\mathrm{g\,cm}^{-3}$ is the mass density of α-pinene and we have used a simple wavelength scaling to convert from the sodium D wavelength $\lambda_{\mathrm{D}} = (589 \pm 1)\,\mathrm{nm}$ to our chosen wavelength $\lambda = (532.0 \pm 0.5)\,\mathrm{nm}$.[3] The measured rotation angles are

$$\Delta\theta'(1S,5S) = (-5.14 \pm 0.11)^\circ$$
$$\Delta\theta'(1R,5R) = (+5.26 \pm 0.11)^\circ,$$

which reveals that the enantiomeric excesses are

$$
\begin{aligned}
ee(1S,5S) &= \frac{\Delta\theta'(1S,5S)}{\Delta\theta(1S,5S)} \\
&= (96.4 \pm 2.1)\% \\
ee(1R,5R) &= \frac{\Delta\theta'(1R,5R)}{\Delta\theta(1R,5R)} \\
&= (98.6 \pm 2.1)\%.
\end{aligned}
$$

It is reasonable that $ee(1S,5S)$ is smaller than $ee(1R,5R)$ as we illuminated the $(1S,5S)$ sample for many hours more than the $(1R,5R)$ sample during the construction and calibration of our instrument, causing more thermal racemization. We note also that the polarization azimuths of the incident light in the scattering volumes are

$$
\begin{aligned}
\theta^{\mathrm{i}}(1S,5S) &= \theta^{\mathrm{i}} + \Delta\theta'(1S,5S)/2 \\
&= (87.43 \pm 0.26)^\circ \\
\theta^{\mathrm{i}}(1R,5R) &= \theta^{\mathrm{i}} + \Delta\theta'(1R,5R)/2 \\
&= (92.63 \pm 0.26)^\circ,
\end{aligned}
$$

where $\theta^{\mathrm{i}} = (90.00 \pm 0.25)^\circ$ is the polarization azimuth of the incident light before entering the samples and we take the scattering volumes to be located in the middles of the cuvettes.

Basic Rayleigh scattering theory gives[2–4]

$$
\begin{aligned}
q(1S,5S) &= 1 + \frac{1}{12}\{1 + \cos[2\theta^{\mathrm{i}}(1S,5S)]\}[45N\chi_T k_B T(\alpha^2/\beta^2) + 1] \\
&= 1.0093 \pm 0.0026 \\
q(1R,5R) &= 1 + \frac{1}{12}\{1 + \cos[2\theta^{\mathrm{i}}(1R,5R)]\}[45N\chi_T k_B T(\alpha^2/\beta^2) + 1] \\
&= 1.0097 \pm 0.0027
\end{aligned}
$$

to leading order, where $N = (3.790 \pm 0.005) \times 10^{27}\,\mathrm{m}^{-3}$ is the molecular number density of α-pinene; $\chi_T = (7.750 \pm 0.005) \times 10^{-10}\,\mathrm{Pa}^{-1}$ is the isothermal compressibility of α-pinene;[1] $T = (293 \pm 10)\,\mathrm{K}$ is the temperature of the illuminated samples; and $\alpha^2/\beta^2 = 50 \pm 10$ is a ratio of isotropic to anistropic rotational invariants for α-pinene, estimated using Gaussian 09.

Putting the above together, we find that the magnitude rescaling factor is

$$f = 1.035 \pm 0.016,$$

giving corrected measured values of

$$\Delta(1S, 5S) = (+3.6 \pm 0.3) \times 10^{-4}$$
$$\Delta(1R, 5R) = (-3.6 \pm 0.3) \times 10^{-4},$$

as quoted in the main text.

References

(1) Pajić, J. I.; Ivaniš, G.; Radović, I.; Grujić, A.; Stajić-Trošić, J.; Stijepović, M.; Kijevčanin, M. Experimental densities and derived thermodynamic properties of pure p-cymene, α-pinene, limonene and citral under high pressure conditions. *J. Chem. Thermodyn.* **2020**, *144*, 106065.

(2) Landau, L. D.; Lifshitz, E. M.; Pitaevskii, P. *Electrodynamics of Continuous Media*, 2nd ed.; Pergamon Press: Oxford, 1984.

(3) Barron, L. D. *Molecular Light Scattering and Optical Activity*, 2nd ed.; Cambridge University Press: Cambridge, 2004.

(4) Cameron, R. P.; Alexakis, E. I.; Arnold, A. S.; McArthur, D. Theory of Rayleigh-

Brillouin optical activity light scattering applicable to chiral liquids. *Phys. Chem. Chem. Phys.* **2024**, *26*, 11641.
